# Chloroxine overrides DNA damage tolerance to restore platinum sensitivity in high-grade serous ovarian cancer

**DOI:** 10.1038/s41419-021-03665-0

**Published:** 2021-04-14

**Authors:** Vera L. Silva, Jayeta Saxena, Francesco Nicolini, Joseph I. Hoare, Stephen Metcalf, Sarah A. Martin, Michelle Lockley

**Affiliations:** 1 Centre for Cancer Cell and Molecular Biology, Barts Cancer Institute, Queen Mary University of London, London, UK; 2 Department of Gynaecological Oncology, Cancer Services, University College London Hospital, London, UK

**Keywords:** Ovarian cancer, High-throughput screening

## Abstract

High-grade serous cancer (HGSC) accounts for ~67% of all ovarian cancer deaths. Although initially sensitive to platinum chemotherapy, resistance is inevitable and there is an unmet clinical need for novel therapies that can circumvent this event. We performed a drug screen with 1177 FDA-approved drugs and identified the hydroxyquinoline drug, chloroxine. In extensive validation experiments, chloroxine restored sensitivity to both cisplatin and carboplatin, demonstrating broad synergy in our range of experimental models of platinum-resistant HGSC. Synergy was independent of chloroxine’s predicted ionophore activity and did not relate to platinum uptake as measured by atomic absorption spectroscopy. Further mechanistic investigation revealed that chloroxine overrides DNA damage tolerance in platinum-resistant HGSC. Co-treatment with carboplatin and chloroxine (but not either drug alone) caused an increase in γH2AX expression, followed by a reduction in platinum-induced RAD51 foci. Moreover, this unrepaired DNA damage was associated with p53 stabilisation, cell cycle re-entry and triggering of caspase 3/7-mediated cell death. Finally, in our platinum-resistant, intraperitoneal in vivo model, treatment with carboplatin alone resulted in a transient tumour response followed by tumour regrowth. In contrast, treatment with chloroxine and carboplatin combined, was able to maintain tumour volume at baseline for over 4 months. In conclusion, our novel results show that chloroxine facilitates platinum-induced DNA damage to restore platinum sensitivity in HGSC. Since chloroxine is already licensed, this exciting combination therapy could now be rapidly translated for patient benefit.

## Introduction

Ovarian cancer is the seventh most common cancer type amongst women^1^ and the most common histological subtype is high-grade serous cancer (HGSC)^2^. Standard treatment is still reliant on combination surgery and platinum-based chemotherapy^3^. Despite this aggressive approach, 85% of patients will subsequently relapse and sequential platinum treatment inevitably results in chemotherapy resistance^4^. Circumventing this pervasive problem is an unmet clinical challenge.

Platinum compounds covalently bind DNA, generating protein–DNA or DNA–DNA complexes, along with inter- and intra-strand adducts^5,6^. This ultimately generates DNA lesions, followed by activation of the DNA damage response (DDR)^5,6^. Apoptosis eventually occurs when damage exceeds repair. Platinum resistance is a multifactorial process that has been extensively investigated^7^. Many potential mechanisms underlie this event, including failure to uptake or accumulate platinum^8^, augmented DNA damage repair machinery^9^, increased tolerance to unrepaired DNA lesions^10^, defects in signal transduction^7^, inactivation of pro-apoptotic events^11^ and upregulation of autophagic pathways^6^. The elucidation of these mechanisms is yet to support the discovery of more efficient treatments for resistant HGSC^3^.

Repurposing non-oncological drugs for clinical applications is an exciting and cost-effective strategy that can provide rapid clinical translation^12^. We have previously developed a unique panel of platinum-resistant cell lines and in vivo models and shown that they closely resemble the genetic, transcriptional and clinical features of human HGSC^13^. Using these novel cell lines, we employed a drug screening approach to identify hit drugs that could potentially reverse platinum resistance. The compound library encompassed 1177 small molecules, 90% of which are marketed drugs, many with unknown anticancer properties. We identified chloroxine, a 8-hydroxy-quinoline (8-HQ) with antibacterial and antifungal activities^14^. To our knowledge, there are no reports regarding the activity of chloroxine as an anticancer agent and very little is known about its inherent cellular mechanism.

Herein, we reveal for the first time, the synergy between chloroxine and platinum agents in resistant HGSC both in vitro and in vivo. We unravel the novel mechanism of action of chloroxine in overriding the inherent tolerance to DNA damage in platinum-resistant cancer cells and facilitating cancer cell death by apoptosis. Moreover, chloroxine showed tumour-static effect in vivo, when used in combination with carboplatin; thus, chloroxine could have significant impact on the treatment of patients with platinum-resistant HGSC.

## Materials and methods

### Cell culture and platinum-resistant cell lines

Human HGSC cell lines OVCAR4 and COV318 were obtained from Prof. Fran Balkwill (Barts Cancer Institute, UK). Platinum-resistant HGSC cell lines (Ov4Cis, Ov4Carbo and CovCis) were generated by serial culture in increasing concentrations of either cisplatin or carboplatin, as we described previously^13^ and subsequently cultured in drug-free media. Cells were grown at 37 °C in a humidified incubator with 95% air and 5% CO_2_ in DMEM (Gibco) supplemented with 10% FBS (Gibco) and 1% penicillin/streptomycin (Gibco). All cell lines underwent 16-locus STR verification (DNA Diagnostics Centre, London, UK: February 2016 and European Collection of Authenticated Cell Lines August 2019) and weekly mycoplasma testing.

### Compound library screen

To screen HGSC cell lines for existing drugs that synergise with carboplatin, we used a 96-well plate cell viability assay based on our previous work^15^. The chemical library encompassed 1177 small molecules, 90% of which were marketed drugs, the remaining 10% being bioactive alkaloids. Platinum-sensitive (OVCAR4) and platinum-resistant (Ov4Carbo) cells were seeded in 96-well plates (10^3^ cells/well). On day 2, cells were exposed to either a library compound or equimolar dimethyl sulfoxide (DMSO) vehicle control. On day 5, cells were re-treated with the same library compound together with either carboplatin (5 µM) or vehicle. In all, 5 µM carboplatin was chosen because this dose resulted in minimal cell death in both cell lines (Supplementary Fig. S1A). Each drug in the compound library was therefore tested alone and in combination with carboplatin in both cell lines, in one well of the 96-well plate per experimental condition. The final compound concentration used was 10 µM, based on our previous work^15^ as a dose in which the effects on cell viability are highly likely to be observed. We expected that many of the initial hit drugs, identified at this drug dose in only one well per experimental condition, would be excluded in subsequent validation experiments (see below).

After 7 days’ continuous culture, cell viability in each well was estimated using a luciferase-based ATP assay (CellTitre-Glo, Promega) according to the manufacturer’s instructions. For each well in the 96-well plate, the effect of treatment was represented as a log2-surviving fraction (s.f.) and expressed relative to the median s.f. for the entire 96-well plate. In each cell line, s.f. was compared between library-treated and vehicle-treated wells and also between wells treated with either library+carboplatin or carboplatin alone (Supplementary Table 1). To identify the selective ability to synergise with carboplatin, we predefined a cut-off of s.f. <−2 following library + carboplatin together with s.f. >−2 for library compound alone. Using these criteria, 11 drugs were considered ‘hits’ and selected for further investigation in validation experiments (Supplementary Table 2).

### Synergy validation experiments

Validation experiments were carried out by plating OVCAR4 and Ov4Carbo cells in 96-well plates (10^3^ cells/well) in triplicate wells per condition. Cells were treated using the same experimental protocol as the initial compound library screen (day 2: hit drug (1–10 μM) or vehicle, day 5: carboplatin (0.01–1000 μM) + either hit drug (same dose as on day 2) or vehicle). Cell viability was again estimated using CellTitre-Glo on day 7. Dose–response curves were generated and IC_50_ calculated using GraphPad Prism v.8.0 (nonlinear regression fit to a five-parameter equation). At least three biological repeat experiments were conducted per potential hit compound and analysed using Calcusyn^®^ software to generate isobolograms where a CI (combination index) >1 indicates synergy, CI = 1 indicates addition and CI < 1 indicates antagonism. All 11 potential hit drugs (Supplementary Table 2) were tested in validation experiments. Chloroxine was selected for further investigation because it was the only drug that demonstrated synergy with platinum chemotherapy specifically in HGSCs with pre-existing resistance.

### Preparation of chloroxine in Intralipid^®^

Chloroxine (5,7-dichloro-8-quinolinol, Sigma-Aldrich) was weighed in amounts of 0 (blank), 10, 30, 50 and 100 mg/kg in separate glass vials. Larger clumps were broken down using a spatula. In total, 15 ml of Intralipid emulsion (Sigma-Aldrich) was added to each glass vial, vortex-mixed for 5 min and bath-sonicated for at least 2 h at 40 °C. A previous report^16^ showed that there was no effect of bath sonication on the stability of these emulsions. Optimum solubility was obtained at 10 mg/kg.

### Murine experiments

OVCAR4 and Ov4Carbo cells were previously modified using lentiviruses to express dual GFP/Luciferase and RFP/Luciferase reporters, respectively^13^. All experiments were conducted under UK government Home Office license (P1EE3ECB4) following Institutional Review Board approval. Cells were inoculated by intraperitoneal (IP) injection into 6-week-old female CD1^*nu/nu*^ mice (Charles River Laboratories) in sterile PBS (5 × 10^6^ cells/200 µl). Tumour growth was monitored weekly via bioluminescence using IP injections of D-Luciferin monopotassium salt (3.7 mg/200 ml, ThermoFisher) in PBS. Light emission was recorded using an IVIS^®^ Spectrum (PerkinElmer). Mice were randomly allocated (by order of cage number and existing earmarking) to treatment groups. In an initial optimisation experiment, mice received either carboplatin (50 mg/kg in 0.2 ml PBS IP weekly, *n* = 10) or PBS control (0.2 ml daily IP, *n* = 5). We then selected a sample size of five mice per group for our first in vivo evaluation of chloroxine-delivered IP in Intralipid. Mice in this second experiment received carboplatin (50 mg/kg in 0.2 ml PBS IP weekly, *n* = 5), chloroxine (10 mg/kg in 0.2 ml Intralipid IP once daily, *n* = 5), combination (daily chloroxine and weekly carboplatin, 0.4 ml IP, *n* = 5) or PBS control (0.2 ml daily IP, *n* = 5). In both experiments, treatment commenced once tumours were established, as determined by our predefined light output cut-offs (average radiance between 10^5^ and 10^7^ p/s/cm^2^/sr). Three mice were excluded for not meeting these parameters (one from the carboplatin group, one from the combination group and once from the PBS control group). The appearance of chloroxine in Intralipid solution (see Fig. 2B) prevented blinding of experimental groups. Treatment continued for 4 weeks. Mice were assessed for weight, general health and accumulation of ascites, and were killed once they reached humane endpoint (defined by the UK Home Office guidelines).

### Platinum uptake

In total, 1 × 10^6^ OVCAR4 and Ov4Carbo cells were plated in 10-cm dishes (Corning) and treated after 24 h with 10 μM chloroxine, 100 μM carboplatin or both drugs combined. Up to 48 h later, cells were washed twice with ice-cold PBS. Cells were scraped into 200 μl lysis buffer (150 mM NaCl, 50 mM Tris, 0.05% SDS and 1% triton). In total, 20 μl lysate was collected for protein quantification (Pierce bicinchoninic acid (BCA) kit (Thermo Scientific)). The remainder was spun (13,000×*g* at 4 °C for 5 min) and the supernatant (whole-cell fraction) collected. Samples were adjusted to 1 ml with 0.5% nitric acid (HNO_3_) to ensure platinum (Pt) solubilisation. Pt content was analysed using atomic absorption spectroscopy, AAS (Varian Spectra AAS 220 FS) with a hollow cathode platinum lamp operated at 10 mA. Platinum absorbance was monitored at 265.9 nm corrected for any background signals, with a slit width of 0.2 nm. Argon was used as the inert gas with a constant flow of 3 ml/min. Elemental platinum standards (20–1000 µg/L) were prepared by serial aqueous dilution using High-Purity Standards platinum standard (1000 µg/ml, Sigma-Aldrich) and carboplatin stock (10 mg/ml). In all, 20 μl samples were injected for three measurements per sample on two dishes per condition in duplicate experiments. Pt concentrations were determined by applying an elemental platinum standard curve and normalised to protein levels.

### Copper complexation

Cu-8-HQ (chloroxine) complexes were prepared as described previously^17^. Two equivalents of chloroxine (20 mg, 0.0935 mmol) were added slowly to a stirred solution of CuCl_2_ (8 mg, 0.0468 mmol) in 10 ml of an ethanolic solution and stirred for over 3 h. To assess cytotoxicity, OVCAR4 and Ov4Carbo cells (1000/well) were plated in 96-well plates in triplicate and treated with cupric chloroxine (10 μM) ± carboplatin as before (see synergy validation, *n* = 3 biological repeats). Cell viability was determined using CellTiter-Glo and dose–response curves generated.

### Copper enrichment assays

To simulate the in vivo copper status of cancer cells, OVCAR4 and Ov4Carbo cells were cultured in media containing 25 μM CuCl_2_ for 1 week, as previously described^18^. Cells (1000/well) were then plated in 96-well plates and treated with chloroxine (10 μM) ± carboplatin in triplicate as before (see synergy validation, *n* = 3 biological repeats). For short-term enrichment, cells were plated in 96-well plates and pre-treated with 10 μM CuCl_2_ for 48 h. Cell viability was determined on day 7 using CellTiter-Glo and dose–response curves generated.

### OPRK1

OVCAR4 and Ov4Carbo cells (1000/well in triplicate) were seeded in 96-well plates (triplicate) and treated with chloroxine (10 μM) ± carboplatin as before (see synergy validation, *n* = 8 biological repeats). The selective κ-opioid agonist: U50,488 ((±)-trans-U-50488 methanesulfonate salt, Sigma-Aldrich) and antagonist: nor-binaltorphimine (nor-BNI, Sigma-Aldrich) were used at 10 μM in place of chloroxine and in combination with carboplatin on day 5. Control plates displaying dose–response curves for each individual compound were seeded in parallel. Cell viability was determined on day 7 using CellTiter-Glo assay and dose–response curves generated.

### Western blot

OVCAR4 and Ov4Carbo (4 × 10^5^ cells/well) were seeded in six-well plates. Cells were washed with PBS and sonicated (Bioruptor Pico-RM 343 for 10 min) in ice-cold lysis buffer (150 mM NaCl, 50 mM Tris, 0.05% SDS and 1% triton), supplemented with 1% protease/phosphatase inhibitor cocktail (Roche). Samples were centrifuged (13,000×*g*, 4 °C, 20 mins) and protein concentration determined (Pierce BCA assay kit (Thermo Scientific)). In total, 30 μg/20 μl protein were prepared in SDS loading buffer, boiled for 5 min, separated on 4–12% Bis–Tris gels (Life Technologies) and transferred to nitrocellulose membranes (GE Healthcare) by semi-dry transfer (Trans-Blot^®^ SD Cell, BioRad). Membranes were blocked in 5% non-fat milk/PBS for 1 h at room temperature and incubated with primary antibodies for p53 (1:1000, Sc-126 Santa Cruz, *n* = 4 biological repeats), RAD51 (1:1000, PA5-27195, Invitrogen, *n* = 3 biological repeats), GADPH (1:2000, Sc-47724 Santa Cruz), Tubulin (1:2000, ab52866 Abcam) and γH2AX (ser139 1:500, JBW301 Millipore, *n* = 8 biological repeats) at 4 °C overnight, followed by incubation with secondary anti-mouse HRP (1:2000, P0260 DAKO) and anti-rabbit HRP (1:1000, P0448 DAKO) for 1 h at room temperature. Proteins were visualised by enhanced chemiluminescence (GE Healthcare), imaged (Amersham Image 600, GE Healthcare) and quantified by densitometry (ImageQuant TL software package).

### Immunofluorescence for DNA damage

OVCAR4 and Ov4Carbo cells (8 × 10^4^ cells/well) were seeded in duplicate on poly-l-lysine-coated coverslips and allowed to adhere overnight. Cells were then treated with chloroxine (10 μM), carboplatin (50 μM or 100 μM) or combination [(chloroxine (10 μM) + carboplatin 50 μM or 100 μM)] for 4 and 24 h. The medium was aspirated and 0.1% Triton (Sigma-Aldrich) in PBS added for 1 min prior to fixing in 3% paraformaldehyde/2% sucrose for 30 min. Cells were then blocked with 3% BSA/PBS for 1 h. Next, staining was performed with antibodies for γH2AX (1:800, Millipore JBW301) and RAD51 (1:1000, Invitrogen PA5-27195) for 40 min at 37 °C. Cells were incubated with secondary antibodies (AlexaFluor-488 and AlexaFluor-568, Invitrogen) for 30 min at 37 °C, and co-stained with DAPI (1:10,000, 1 mg/ml Sigma). Coverslips were mounted with Mowiol and images captured using a Zeiss 710 confocal microscope. Foci were counted and scored manually using ImageJ software. At least 100 nuclei were counted for each condition (using duplicate plate wells) and cells with >5 foci/nucleus were considered positive (three biological replicates were conducted).

### Flow cytometry (cell cycle)

Cells were treated with chloroxine (10 μM), carboplatin (100 μM) or the combination (chloroxine 10 μM + carboplatin 100 μM) and triplicate samples were harvested 24 h and 48 h post treatment in two biological repeat experiments. Cells were centrifuged (1000 × *g* for 5 min), washed three times with cold PBS and fixed in 70% (v/v) ethanol/PBS. Next, cells were re-suspended in 0.25 ml PBS containing 25 μg RNase and 12.5 μg propidium iodide. Following 30 min of incubation at 37 °C, cell cycle profiles were acquired with LSR Fortessa^TM^ (BD Biosciences), using a 670-nm long-pass filter and counting 10,000 cells per sample. Data were analysed using FlowJo v8.

### Caspase 3/7 assay

The synergy validation protocol detailed above was used to determine caspase 3/7 activity. Briefly, 1000 cells were plated, in triplicate, in 96-well plates and treated with chloroxine (1–50 μM), carboplatin (10–300 μM) or with the combination. Caspase 3/7 activity was determined using Caspase-Glo^®^ 3/7 Assay System (Promega) up to 7 days later. Duplicate plates were seeded to determine cell viability using CellTiter-Glo and three biological repeats were carried out.

### Statistics

All data were presented as mean ± s.d. and analyses were performed using GraphPad Prism 8.0. Groups were compared to establish comparable variance and statistical significance was calculated using a two-tailed unpaired *t* test, one-way ANOVA (followed by post hoc Kruskal–Wallis test) or two-way ANOVA (followed by Tukey post hoc test) for group analysis and defined as follows: *P* < 0.0001 (****), *P* < 0.001 (***), *P* < 0.01 (**), *P* < 0.05 (*) or *P* > 0.05 (ns). *n* refers to biological replicates and the established scientific standard of *n* ≥ 3 was applied throughout.

## Results

### Chloroxine synergises with carboplatin in platinum-resistant HGSC in vitro

**Fig. 1:**
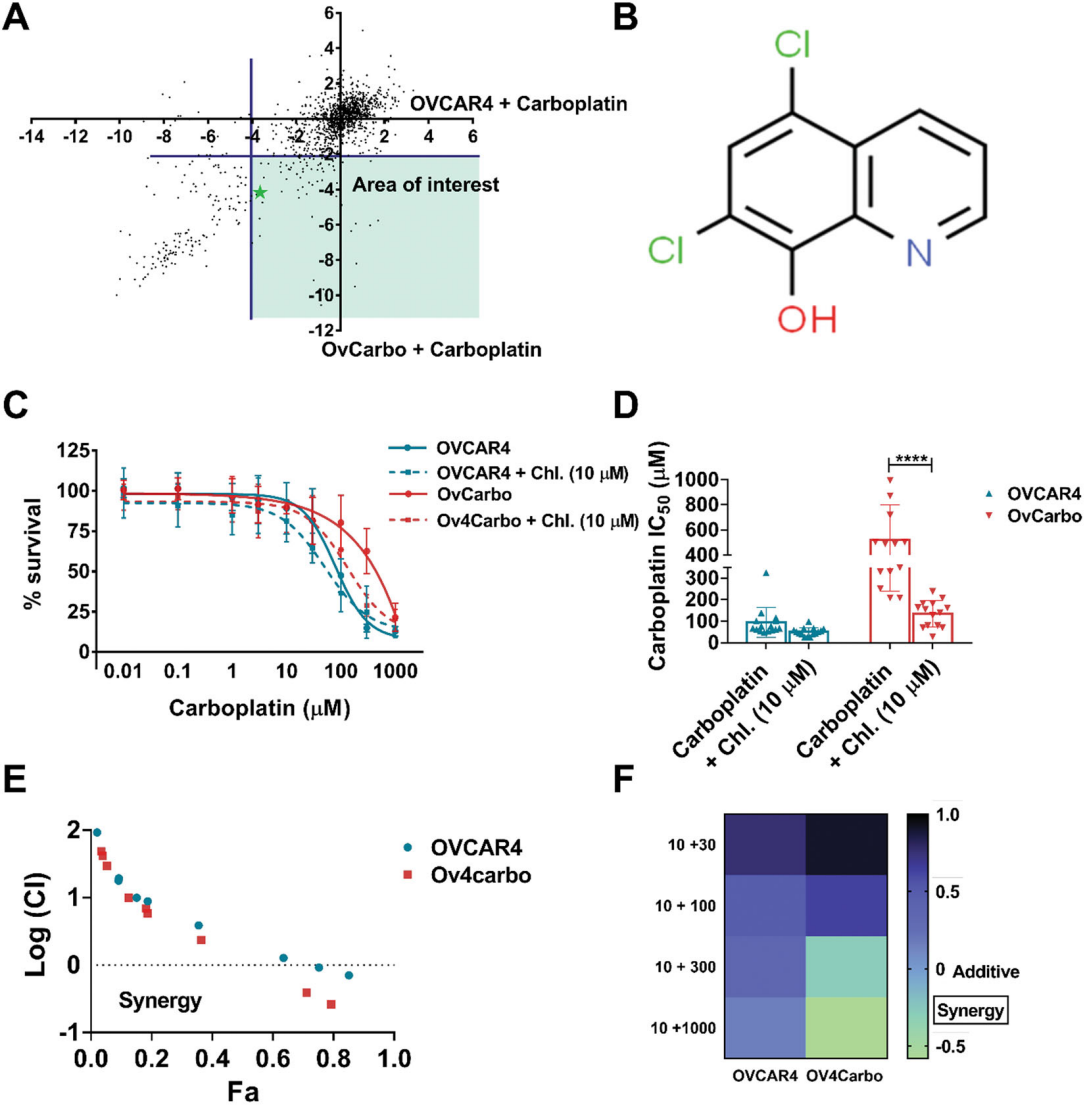
Drug screen and in vitro synergy between chloroxine (Chl. 10 µM) and carboplatin on the viability of OVCAR4 (sensitive) and Ov4Carbo (resistant) ovarian cancer cells. **A** z-score (surviving fraction, s.f.) plot evidencing drug screen output. Each black dot represents one small-molecule drug in the library. Potential hits are highlighted in the area of interest (green star indicates target drug: chloroxine). **B** Chemical structure of 5,7-dichloro-8-quinolinol (chloroxine). **C** Carboplatin dose response. **D** IC_50_ plot. **E** Fa–CI plot or Chou–Talalay displaying combination index (in log) vs. fraction of affected cells (Fa); **F** heatmap demonstrating synergy in multiple biological repeats (*n* = 13). Cell viability was determined using CellTiter-Glo^®^. IC_50_ was calculated using GraphPad Prism v.8.3.0. mean ± s.d, unpaired *t* test, ****P < 0.0001. The combination index (CI) was determined using Calcusyn software, where values <1 (log 0) reflect drug synergy.

We employed a library of 1177 compounds to identify drugs that can restore sensitivity to carboplatin in HGSC^15^. Cell viability was assessed using CellTiter-Glo^®^ and the effect of each compound was determined by comparing luminescence output following drug, carboplatin, drug + carboplatin and vehicle treatment in OVCAR4 and Ov4Carbo cells. This effect was represented as a log_2_-surviving fraction (s.f.) and expressed relative to the median s.f. for the entire 96-well plate (Fig. 1A and Supplementary Table 1). In total, 11 potential “hit” compounds were identified that fulfilled our predefined criteria of s.f. <−2 following library + carboplatin together with s.f. >−2 for library compound alone (Supplementary Table 2). All 11 compounds were tested in validation experiments and only chloroxine (Fig. 1B) was validated as a “hit”.

Chloroxine potentiated the effect of carboplatin in Ov4Carbo (carboplatin-resistant) cells in multiple repeat experiments (Fig. 1C, D). To determine if this combination was additive or synergistic, we analysed individual and combinatorial drug effects using CompuSyn software^19^. Chou–Talalay isobolograms (Fig. 1E) showed that the combination of chloroxine and carboplatin was synergistic at chloroxine doses of 10 μM and above (Supplementary Fig. S1), and that synergy was superior in carboplatin-resistant compared to -sensitive cells (Fig. 1F). These data strongly support chloroxine as a candidate drug to restore carboplatin sensitivity in HGSC. Importantly, the striking synergistic effect of chloroxine was also observed with cisplatin in additional platinum-resistant HGSC cell lines, including Ov4Cis (Supplementary Fig. S2A, B) and CovCis (Supplementary Fig. S2C, D).

### Chloroxine stabilises tumour burden in carboplatin-resistant intraperitoneal xenografts

**Fig. 2:**
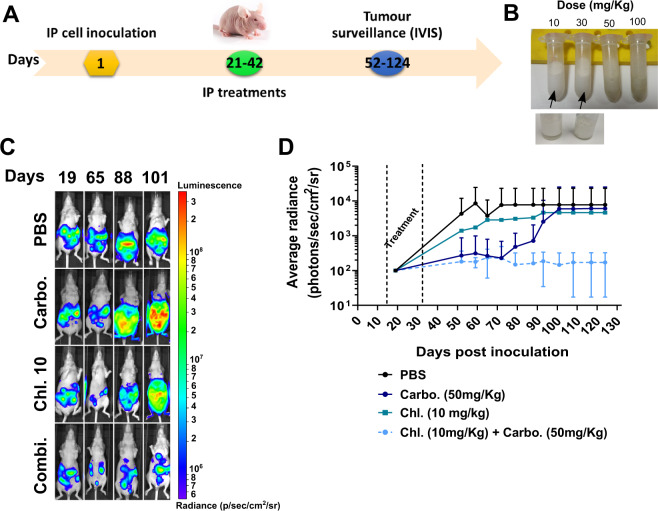
In vivo activity for the combination of chloroxine (Chl. 10 mg/kg) and carboplatin (50 mg/kg) in intraperitoneal Ov4Carbo xenografts. **A** Schematic of in vivo schedule, including treatment and bioluminescence imaging timeframe. **B** Representative images highlighting maximum solubility of chloroxine (≤30 mg/kg) in intralipid 20%. **C** Representative bioluminescence images pre- and post treatment and light emission over time. IP xenografts were created in female CD1^*nu/nu*^ mice by injecting Ov4Carbo-Luc (5 × 10^6^ cells per mouse). **D** Data depict different treatment groups showing mean ± s.d., *n* = 4–14.

To assess chloroxine’s activity in vivo, clinically relevant HGSC orthotopic intraperitoneal models were created, as we previously described^13^. The predetermined in vivo treatment and tumour surveillance schedule are depicted in Fig. 2A. Chloroxine solubility was limited in saline and so was prepared instead in a 20% intralipid solution (as previously described for other hydroxyquinolines)^20^ at concentrations equivalent to 10, 30, 50 and 100 mg/kg. Well-solubilised solutions were observed up to 30 mg/kg (Fig. 2B).

HGSC IP xenografts were then created in female nude mice (day 0) using Ov4Carbo-resistant cells expressing firefly luciferase (Luc). Tumour growth was monitored with weekly bioluminescence imaging (BLI), and mice were killed when they reached humane endpoints pre-defined by the UK Home Office. Mice bearing established tumours on day 19 (average radiance 10^5^–10^7^p/s/cm^2^/sr) (Fig. 2C) were randomly allocated into four treatment groups on day 21. Animal numbers were insufficient to compare survival between the treatment groups. However, we observed that chloroxine-treated mice depicted a steady increase in tumour growth over time, comparable to that of the vehicle-treated group (Fig. 2C, D). Ov4Carbo-Luc xenografts initially responded to carboplatin, but subsequently developed resistance to treatment and light emission increased from day 70 onwards, such that it overlapped with vehicle-treated control mice. In contrast, when chloroxine was used in combination with carboplatin, light output was maintained at baseline (average radiance below 10^3^p/s/cm^2^/sr) for the duration of the experiment (Fig. 2C, D). Thus, the combination of chloroxine and carboplatin had an overall tumour-static effect (average tumour radiance at endpoint 1.71 × 10^2^ p/s/cm^2^/sr) compared to the carboplatin monotherapy group (average tumour radiance at endpoint 6.07 × 10^3^p/s/cm^2^/sr).

### Chloroxine binds copper, but carboplatin synergy is independent of its ionophore activity

**Fig. 3:**
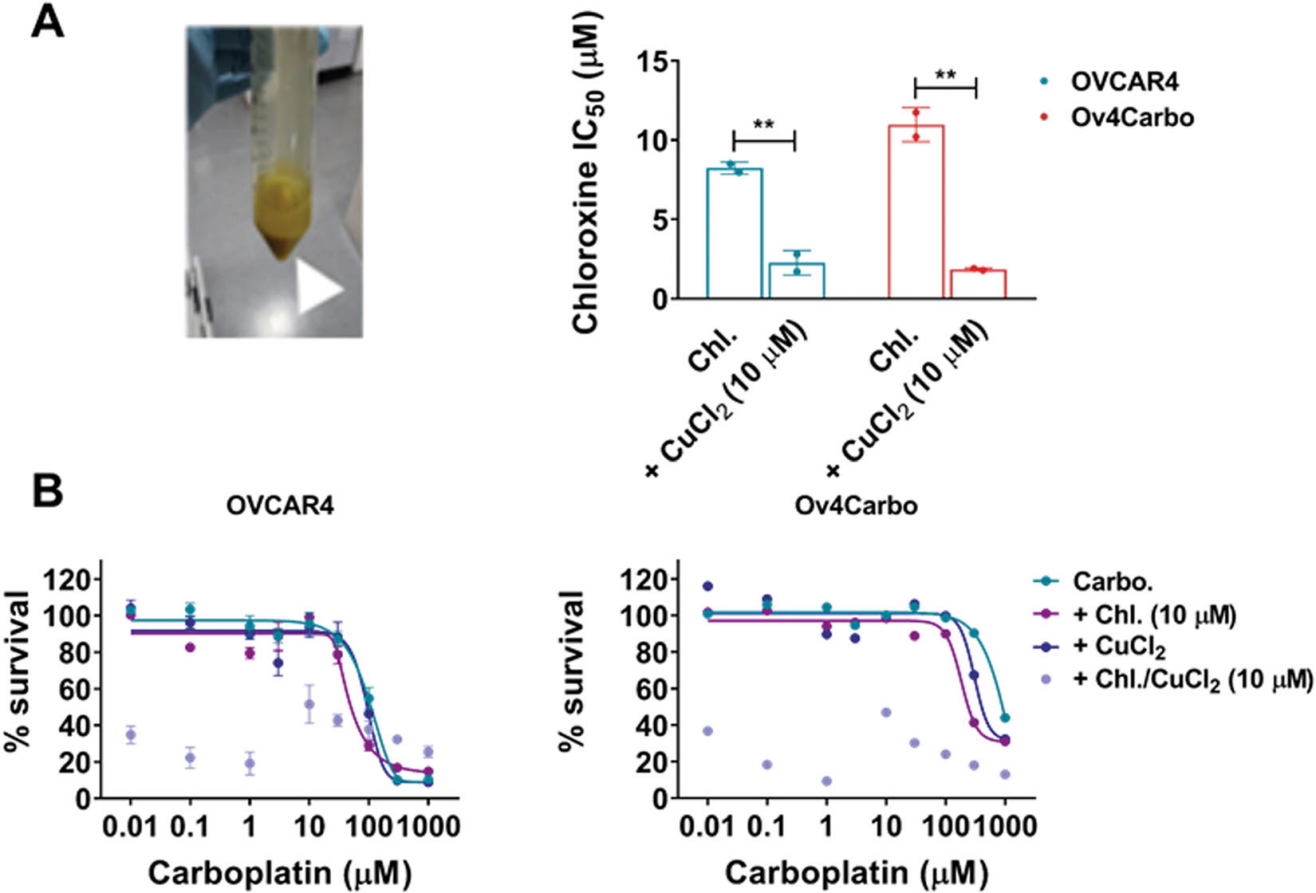
Role of copper (CuCl_2_) complexed with chloroxine in mediating carboplatin/chloroxine synergy in OVCAR4 (sensitive) and OV4Carbo (resistant) HGSC cells. **A** IC_50_ plot for chloroxine (Chl) or chloroxine/CuCl_2_ complex (+CuCl_2_ 10 μM). The inset shows representative image of chloroxine mixed in a 2:1 molar ratio with CuCl_2_ in an ethanolic solution. The appearance of intensified yellow colour indicated formation of a copper complex and **B** carboplatin + chloroxine/CuCl_2_ complex (10 μM). Cell viability was determined using CellTiter-Glo^®^. IC_50_ was calculated using GraphPad Prism v.8.3.0. Mean ± s.d, *n* = 3, unpaired *t* test, **P < 0.01.

8-hydroxyquinolines depict strong metal (Cu^2+^ and Zn^2+^) binding activity^17,20,21^ that leads to proteasome inhibition^22^, lysosome destabilisation^23^, ROS generation and ultimately cell death. To investigate whether chloroxine can also exhibit such activity, a chloroxine solution was mixed in a 2:1 molar ratio with CuCl_2_. An intense colour change was observed^18^, confirming the chemical reaction typical for metal complex binding (Fig. 3A, inset). We then sought to determine if this cupric-chloroxine complex had relevant in vitro activity. When cells were treated with the copper–chloroxine complex for 72 h, potentiation of chloroxine cytotoxicity was observed (Fig. 3A), confirming that chloroxine can act as an ionophore. However, when the copper–chloroxine complex was used in combination with carboplatin, we observed a dramatic reduction in cell viability. Raw luminescence units following treatment with the copper–chloroxine complex were extremely low (<2000) preventing further interpretation of any difference between the resulting normalised values (Fig. 3B). These experiments demonstrate that this complex behaves as a new cytotoxic metal compound rather than mimicking chloroxine’s synergistic interaction with carboplatin.

**Fig. 4:**
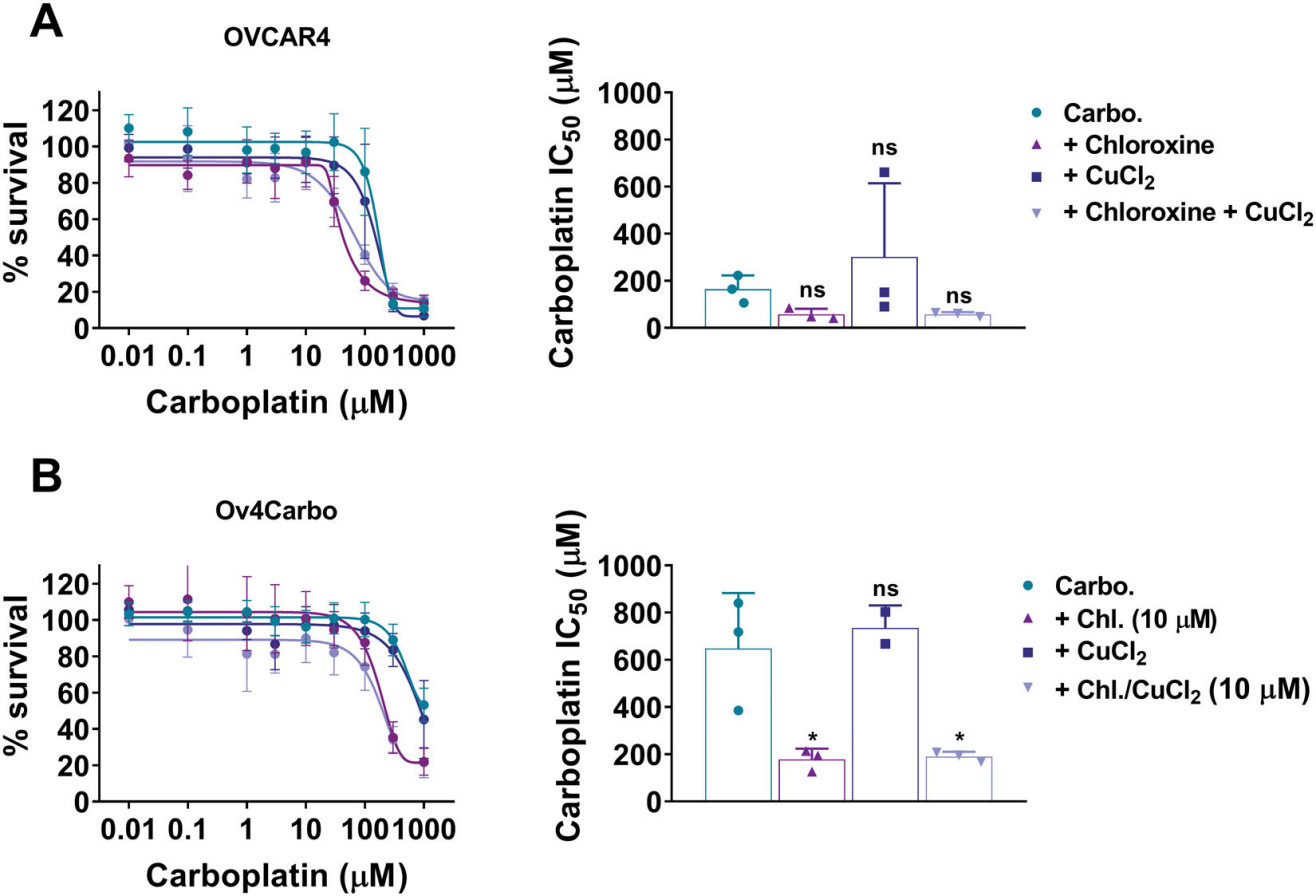
Role of copper (CuCl_2_)-enriched media in mediating carboplatin/chloroxine synergy in OVCAR4 (sensitive) and OV4Carbo (resistant) HGSC cells. For enrichment assays, dose–response and IC_50_ plot for carboplatin + chloroxine (10 μM) were determined in media enriched with CuCl_2_ (10 μM) for 48 h, in **A** OVCAR4 and **B** Ov4Carbo cells. Cell viability was determined using CellTiter-Glo^®^. IC_50_ was calculated using GraphPad Prism v.8.3.0. Mean ± s.d, *n* = 3, group analysis was performed using one-way ANOVA with post hoc Kruskal–Wallis test, *P < 0.05, ns not significant.

To further investigate whether this ionophore activity could be responsible for the synergy we had observed, we reproduced a more physiologically relevant copper environment in vitro by culturing cells in copper-enriched media (10 μM) for 48 h prior to CellTiter-Glo assays. Although the synergistic interaction of chloroxine and carboplatin was once again verified, copper enrichment failed to alter the activity of carboplatin alone and did not influence its interaction with chloroxine in both OVCAR4 (Fig. 4A) and Ov4Carbo cells (Fig. 4B). We also verified that CuCl_2_ alone was non-toxic (Supplementary Fig. S3A) and contrary to the complexation assays (Fig. 3A), copper-enriched media did not alter the toxicity profile of chloroxine (Supplementary Fig. S3B, C). Furthermore, enrichment assays using higher copper concentrations (25 μM and 100 μM) in short- (48 h) and long-term supplementation (7 days) assays also failed to show any effect on drug synergy (data not shown). These experiments imply that chloroxine-mediated ionophore activity is unlikely to be responsible for the synergy with carboplatin that we had observed.

### Chloroxine does not alter platinum uptake or OPRK1 activity in vitro

Platinum uptake is mediated by both passive diffusion and active transporters, and cells that fail to successfully accumulate platinum, either by reduced import or excessive export, have been shown to develop resistance^8^. We measured whole-cell concentrations of platinum using AAS (atomic absorption spectroscopy) after drug treatment. Based on previous experimental procedures^24^, we found that cell pellets treated with lysis buffer containing 0.05% SDS, 1% triton and 0.5% HNO_3_ yielded excellent recovery of platinum (Pt) signal (Supplementary Fig. 5A). Standard curves confirmed the validity and linearity of our assay (*Y* = 0.00187–0.054, *R*^2^ = 0.9908) by showing that carboplatin stock standards yielded equal Pt signal to that of gold-standard Pt stocks. After carboplatin treatment, platinum uptake was observed in both OVCAR4 and Ov4Carbo cells, while vehicle- and chloroxine-treated samples maintained baseline, background signal for the duration of the experiment (Supplementary Fig. 5B, C). There was no significant difference in platinum uptake between the two cell lines. Further, the combination of chloroxine and carboplatin did not influence Pt uptake, suggesting that synergy is not dependant on intracellular Pt accumulation.

**Fig. 5:**
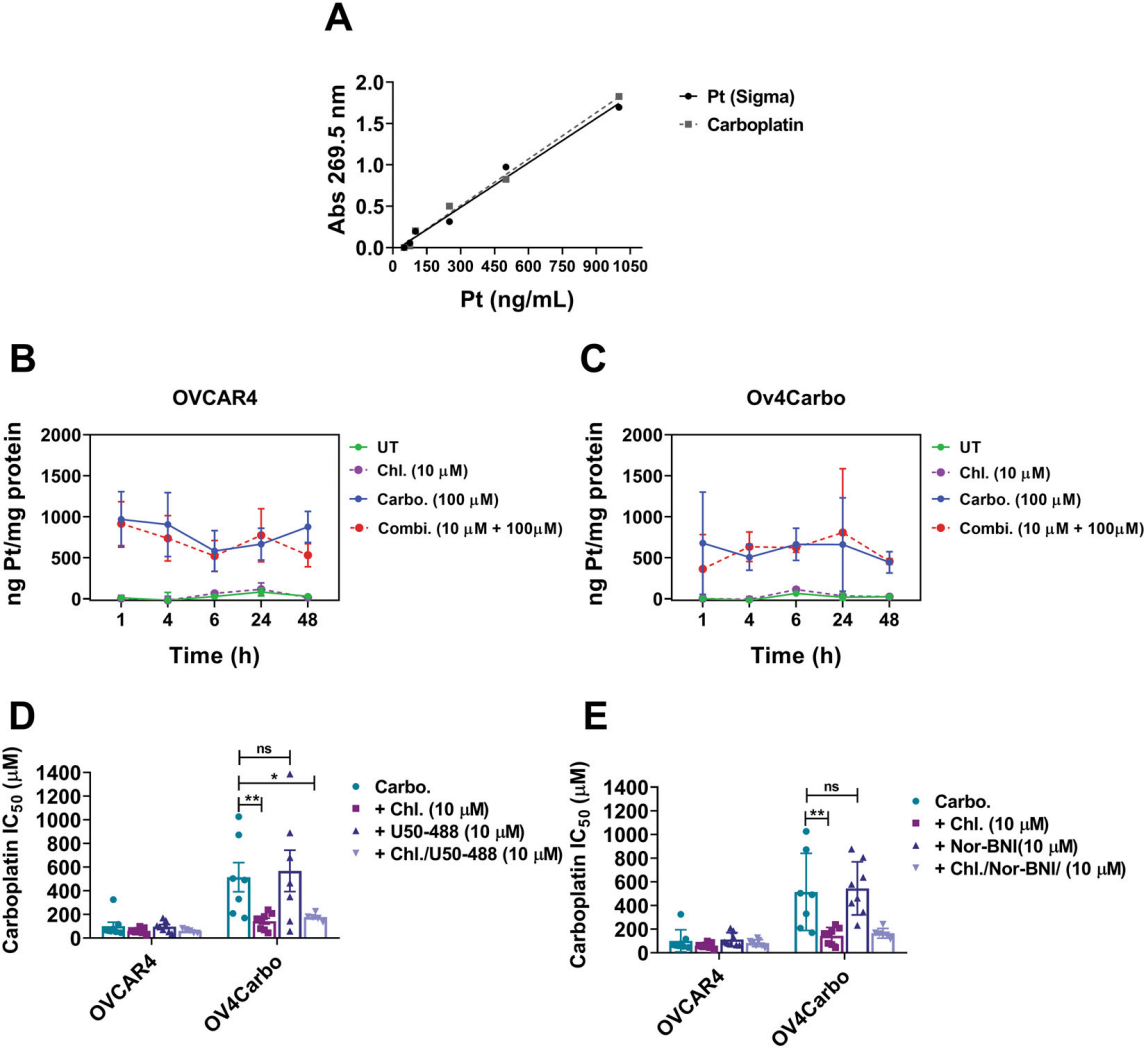
Analysis of platinum uptake and OPRK1-mediated synergy in OVCAR4 (sensitive) and Ov4Carbo (resistant) ovarian cancer cells. **A** Standard curve generated from platinum gold standard (sigma) and carboplatin stock; comparison of platinum accumulation over time after incubation with chloroxine (10 µM), carboplatin (100 µM) and combination of both drugs in (**B**) OVCAR4 and (**C**) Ov4Carbo. Pt quantity was determined by AAS and normalised to total protein. IC_50_ plots for (**D**) OPRk1 agonist (U50-488, 10 µM) and (**E**) antagonist (nor-BNI, 10 µM), when used in combination with carboplatin + chloroxine. Cell viability was determined using CellTiter-Glo^®^. IC_50_ and linear regression (*Y* = 0.00187–0.054, *R*^2^ = 0.9908) was calculated using GraphPad Prism v.8.3.0. AAS assays: mean ± s.d., *n* = 3; agonist/antagonist assays: mean ± s.d., *n* = 8, unpaired *t* test, **P < 0.01, *P < 0.05, ns not significant.

Two previous reports have described a possible function of chloroxine as an inhibitor of the kappa opioid receptor, OPRK1^25,26^. To explore whether synergy between chloroxine and carboplatin was mediated by OPRK1, we repeated our established synergy assays with the addition of an OPRK1 agonist (U50, 488) and antagonist (nor-BNI). U50,488 (10 μM) did not alter carboplatin sensitivity (Fig. 5D, dark-blue bar) and failed to reverse chloroxine’s synergistic effect (Fig. 5D, light-grey bar). In addition, the selective antagonist, nor-BNI^27^, also did not alter the dose response to carboplatin (Fig. 5E, dark-blue bar), and had no effect on chloroxine’s synergy with carboplatin (Fig. 5E, light-grey bar). Taken together, these compounds did not affect the cytotoxicity for carboplatin or the synergy we observed between carboplatin + chloroxine (10 μM) (see also Supplementary Fig. S4), implying that chloroxine was not potentiating the effect of carboplatin through OPRK1-mediated signalling pathways.

### Chloroxine in combination with carboplatin regulates the γ-H2AX–RAD51 axis and stabilises p53 in platinum-resistant cells

We next evaluated the formation of DNA double-strand breaks (DSB) and repair using the gold-standard markers, γH2AX and RAD51^28^. Cells with >5 foci/nucleus were considered positive. Unsurprisingly, our IF data showed that carboplatin induced high levels of γH2AX but very infrequent RAD51 foci in OVCAR4 cells (Supplementary Fig. S5A, B) consistent with their known platinum sensitivity. Importantly, in this cell line, neither γH2AX nor RAD51 foci were altered by chloroxine either alone or in combination with carboplatin.

**Fig. 6:**
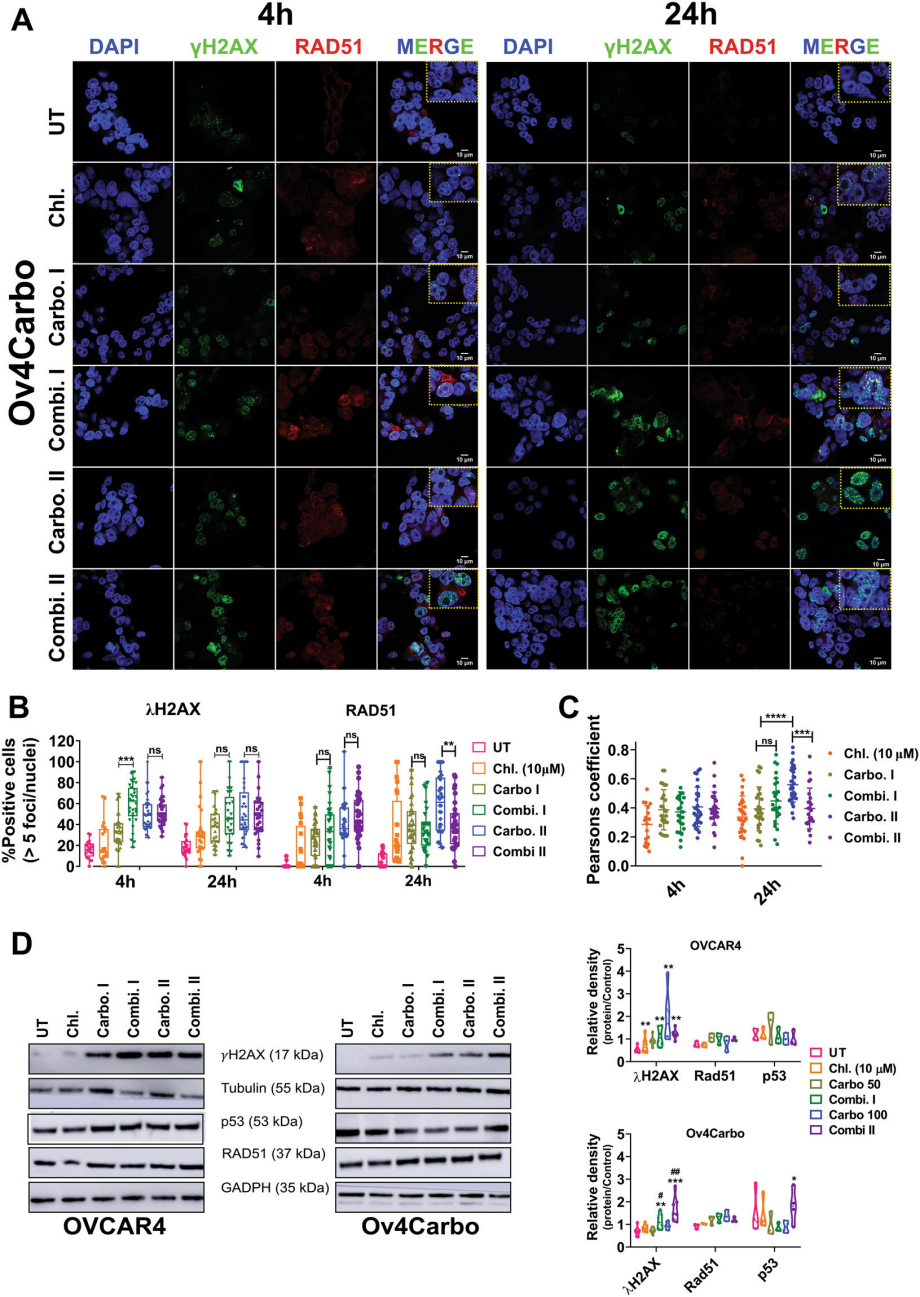
Subcellular analysis of γH2AX, RAD51 and p53 as markers of DNA damage/repair in Ov4Carbo (resistant) ovarian cancer cells. **A** Representative immunofluorescent staining of γH2AX and RAD51, **B** foci quantification and **C** dot plot for Pearson’s correlation coefficients representing co-localisation analysis after 4 and 24 h of treatment. (**D**) Representative immunoblots and densiometric analysis (normalised to tubulin or GADPH), depicting γH2AX phosphorylation (Ser139), p53 and RAD51 expression. Cells were treated with chloroxine (10 μM), carboplatin I (50 μM), combi. I (chloroxine 10 μM + carboplatin 50 μM), carbo II (100 μM) or combi. II (chloroxine 10 μM + carboplatin 100 μM). Untreated cells were used as control. Quantification was expressed as percentage of cells that showed more than five γH2AX or RAD51 foci/nuclei at each time point. Data show mean ± s.d., of individual ROI of duplicate coverslips. At least 100 cells were quantified for each independent experiment (IF and western blot: *n* ≥ 3 biological repeats). IF: two-way ANOVA with Tukey post hoc test; ****P < 0.0001, ***P < 0.001, **P < 0.01 and *P* > 0.05 (ns). WB: unpaired *t* test, ***P < 0.001 **P < 0.01, *P < 0.05 (compared to untreated) and ^##^*P* < 0.01, ^#^*P* < 0.05 compared to carboplatin treated at each time point.

Single-agent chloroxine again did not alter γH2AX foci in Ov4Carbo cells. However, all treatments, including chloroxine alone, carboplatin alone and the combination, induced RAD51 foci more effectively in Ov4Carbo than in OVCAR4. This enhanced DNA repair capacity could contribute to platinum resistance in these cells. In direct contrast to OVCAR4 cells, by 4 h, combined chloroxine and carboplatin treatment of Ov4Carbo cells had induced more γH2AX foci than carboplatin alone, although this was only observed with the lower dose of 50 μM carboplatin (*P* < 0.0001). This was followed by a significant decline in RAD51 at 24 h (*P* = 0.0064) (Supplementary Fig. 6A, B). These data were confirmed by co-localisation analysis (Fig. 6C), which showed a significant reduction in co-localised RAD51/γH2AX foci in Ov4Carbo, 24 h after combined chloroxine and carboplatin treatment compared to carboplatin alone.

These results were then corroborated by immunoblotting, where OVCAR4 cells again expressed significantly higher levels of γH2AX after treatment with carboplatin (*P* < 0.01 compared to untreated cells) with no additional change following combination treatment (ns) (Fig. 6D). In contrast, Ov4Carbo cells depicted low levels of γH2AX expression after exposure to carboplatin alone, but again co-treatment with chloroxine significantly potentiated the expression of γH2AX at both carboplatin doses (chloroxine 10 μM + carboplatin 50 μM: *P* = 0.0111 and chloroxine 10 μM + carboplatin 100 μM, *P* = 0.0012 compared to carboplatin treatment alone). Despite the changes we had observed in RAD51 localisation by IF, total expression of RAD51 by western blot was stable over time and comparable in the two cell lines (Fig. 6D). There was also no significant difference in basal p53 expression between the two cell lines (*P* = 0.08). We did, however, observe increased p53 protein following combination treatment of resistant Ov4Carbo cells (chloroxine 10 μM + carboplatin 100 μM, *P* = 0.0318) but not sensitive OVCAR4 cells (Fig. 6D). Together, our results show that chloroxine co-treatment selectively facilitated carboplatin-induced DNA damage in platinum-resistant cancer cells.

### Chloroxine + carboplatin abrogates G2/M cell cycle arrest and triggers caspase 3/7-mediated cell death

**Fig. 7:**
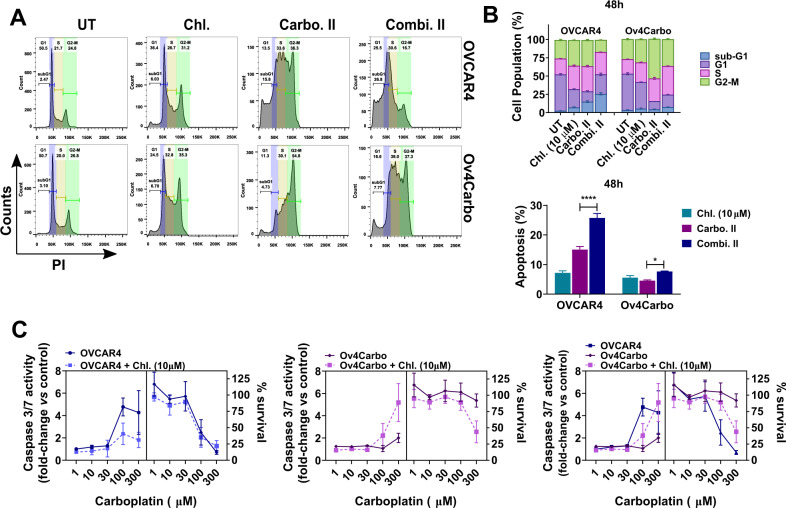
Analysis of cell cycle profile and cell death in OVCAR4 (sensitive) and Ov4Carbo (resistant) ovarian cancer cells. **A** Representative histograms 48 h post treatment; **B** cell cycle distribution (top) and percentage of sub/G1 apoptotic cells (bottom); **C** analysis of caspase 3/7 activity vs. cell survival in OVCAR4 (left panel), Ov4Carbo (middle panel) and summary of assay (right panel). Cells were treated with chloroxine (10 μM), carbo II (100 μM) or combi. II (chloroxine 10 μM + carboplatin 100 μM). Untreated cells were used as control. Data show mean ± s.d., and percentage of G1 (2n), S and G2/M (4n) fraction population (FACs: *n* = 2; caspase 3/7: *n* = 3 biological repeats). Unpaired *t* test, ****P < 0.0001, *P < 0.05 (compared to carboplatin treated).

To elucidate the downstream effects of DNA damage and p53 stabilisation, we analysed cell cycle and cell death following carboplatin and chloroxine co-treatment. Carboplatin treatment caused OVCAR4 cells to arrest in the S phase at both 24 (Supplementary Fig. S6) and 48 h (Fig. 7A, B), with concomitant accumulation of cells in subG1. In contrast, Ov4Carbo cells progressed through the cell cycle into G2/M ( > 50% of the population at 48 h), despite platinum treatment (Fig. 7A, B). Interestingly, in Ov4Carbo cells, this carboplatin-induced G2/M arrest was abrogated after co-treatment with chloroxine such that cell cycle profile matched that obtained when OVCAR4 cells were treated with carboplatin alone. Together, this suggests that chloroxine could restore the capacity of resistant cells to regulate cell cycle and induce apoptosis following carboplatin treatment. To further investigate this, we quantified caspase 3/7 activity. When OVCAR4 cells were treated with either carboplatin or the combination of chloroxine and carboplatin, an increase in caspase 3/7 activity was observed together with an expected decrease in cell survival (Fig. 7C, left panel). There was no difference in caspase 3/7 activity or cell survival in this cell line when carboplatin and the chloroxine/carboplatin combination were compared. In contrast, when Ov4Carbo cells were co-treated with carboplatin and chloroxine, caspase 3/7 activity showed a striking twofold to fivefold increase compared to carboplatin alone (Fig. 7C, middle panel), correlating with the decrease in cell survival and re-sensitisation to carboplatin that we had repeatedly observed. Together, the addition of chloroxine allowed cell cycle re-entry and potentiated carboplatin-induced cell death in platinum-resistant HGSC cells.

## Discussion

Despite HGSC’s initial high response rate to platinum chemotherapy, patients eventually succumb to the disease after developing gradual resistance to therapy. New drugs that can circumvent resistance could have important clinical implications. We show here for the first time that the drug chloroxine has a strong synergistic effect with both carboplatin and cisplatin in a range of HGSC cell lines and in vivo models.

Chloroxine is a 8-HQ derivative. This family of drugs have the potential to form metal complexes via copper and zinc binding^18,20,21^, acting as proteasome inhibitors^22^ or triggering apoptosis^23,29^. Although we confirmed the ionophore activity of chloroxine, neither copper complexation nor intracellular uptake of either copper or platinum appeared to mediate the re-sensitisation to platinum that we observed. Equally, we were unable to demonstrate that a predicted pharmacological function for chloroxine as an inhibitor of kappa opioid receptors accounted for drug synergy.

HGSC is characterised by genomic instability with pervasive *TP53* mutation^30–32^. Homologous recombination and repair dysfunction is also critical in the pathogenesis of this disease^33^, and cellular responses to carboplatin-induced damage are highly dependent on the proficiency of cell cycle and DDR pathways^5,7^. The status of *TP53* has generated controversy around drug response in vitro^34^; thus, it is imperative to use clinically relevant HGSC models such as OVCAR4^35^, in order to understand the relationship of *TP53* to mechanisms, including the DDR. Over 60% of *TP53*-associated mutations are missense mutations^36,37^, which have been described for OVCAR4 cells^37,38^ and are commonly associated with gain-of-function properties. We show here that chloroxine may well interfere with the DDR, and that stabilisation of p53 in otherwise mutant cells is linked to cell death.

Our data are comparable to other reports^7,39–41^, showing that sensitive OVCAR4 cells can stall DNA replication in G1/S phase in response to single-agent platinum treatment. In addition, OVCAR4 cells exhibited minimal RAD51 focus formation, which could potentially indicate a reduced DNA repair capacity and thus higher sensitivity of OVCAR4 cells to platinum drugs. Although there was no difference in total RAD51 protein expression between the two cell lines, Ov4Carbo cells were able to induce RAD51 foci in response to platinum treatment. These enhanced DNA repair dynamics may explain the relative platinum resistance of Ov4Carbo cells and matches with our previous RNASeq analysis that revealed an increase in the DNA recombination pathway in Ov4Carbo compared to OVCAR4 cells^13^.

Chloroxine treatment alone had minimal impact on γH2AX and RAD51 foci in either cell line. Platinum-sensitive OVCAR4 cells were able to efficiently respond to single-agent carboplatin treatment by increasing γH2AX foci formation at all carboplatin doses and time points tested. In these platinum-sensitive cells, the addition of chloroxine did not increase γH2AX above the high γH2AX activation that had already been achieved with carboplatin alone. In contrast, platinum-resistant Ov4Carbo cells did not induce γH2AX foci in response to the lower dose of 50 µM carboplatin, but the addition of chloroxine was able to override this apparent resistance to carboplatin-induced DNA damage and significantly enhance γH2AX accumulation compared to single-agent carboplatin treatment. Importantly, the very high carboplatin dose of 100 µM was able to induce γH2AX foci even in resistant Ov4Carbo cells and once this had been achieved, chloroxine again did not augment γH2AX any further. It is interesting to note that by western blot, we did detect significantly elevated γH2AX protein in Ov4Carbo cells following co-treatment with chloroxine at both carboplatin doses, perhaps reflecting different sensitivity of these two assays. Induction of γH2AX foci by combination treatment of Ov4Carbo cells was followed by a decrease in RAD51 recruitment. This accumulation of DNA damage would be expected to result in the p53 stabilisation, cell cycle stalling in G1/S and apoptotic cell death that we observed^42,43^. Treatments that can reactivate mutant p53 protein have been shown to potentiate chemotherapy and induce apoptosis^44^, suggesting that chloroxine may exhibit a similar activity^45^.

In conclusion, we have demonstrated that the non-oncological drug chloroxine potentiates the cytotoxicity of carboplatin in resistant subtypes of HGSC. Chloroxine synergy was extended to different platinum agents and a range of cell lines and showed a strong tumour-static effect in vivo. Our data suggest that this synergy is mediated via the persistence and dysfunctional repair of platinum-induced damage, along with p53 stabilisation. We, therefore, propose that chloroxine warrants further investigation as an exciting new combination therapy to target resistant subtypes of HGSC.

## Supplementary information

Supplementary Figures and Legends

Supplementary Table 1

Supplementary Tables and Legends

## References

[1] Zhang Y (2019). Global patterns and trends in ovarian cancer incidence: age, period and birth cohort analysis. BMC Cancer.

[2] McCluggage WG (2011). Morphological subtypes of ovarian carcinoma: a review with emphasis on new developments and pathogenesis. Pathology.

[3] Gadducci A (2019). Current strategies for the targeted treatment of high-grade serous epithelial ovarian cancer and relevance of BRCA mutational status. J. Ovarian Res..

[4] Bowtell DD (2015). Rethinking ovarian cancer II: reducing mortality from high-grade serous ovarian cancer. Nat. Rev. Cancer.

[5] Binju M (2019). Mechanisms underlying acquired platinum resistance in high grade serous ovarian cancer—a mini review. Biochimica et. Biophysica Acta.

[6] Ren JH (2010). Acquired cisplatin resistance in human lung adenocarcinoma cells is associated with enhanced autophagy. Cancer Biother Radiopharm..

[7] Galluzzi L (2012). Molecular mechanisms of cisplatin resistance. Oncogene.

[8] Loh SY, Mistry P, Kelland LR, Abel G, Harrap KR (1992). Reduced drug accumulation as a major mechanism of acquired resistance to cisplatin in a human ovarian carcinoma cell line: circumvention studies using novel platinum (II) and (IV) ammine/amine complexes. Br. J. Cancer.

[9] Fink D, Aebi S, Howell SB (1998). The role of DNA mismatch repair in drug resistance. Clin. Cancer Res..

[10] Roos WP (2009). The translesion polymerase Rev3L in the tolerance of alkylating anticancer drugs. Mol. Pharmacol..

[11] Gadducci A, Cosio S, Muraca S, Genazzani AR (2002). Molecular mechanisms of apoptosis and chemosensitivity to platinum and paclitaxel in ovarian cancer: biological data and clinical implications. Eur. J. Gynaecol. Oncol..

[12] Corsello SM (2020). Discovering the anticancer potential of non-oncology drugs by systematic viability profiling. Nat. Cancer.

[13] Hoare J. I. et al. Platinum resistance induces diverse evolutionary trajectories in high grade serous ovarian cancer. Preprint at https://www.biorxiv.org/content/10.1101/2020.07.23.200378v1 (2020).

[14] Shahabadi N, Zendehcheshm S (2020). Evaluation of ct-DNA and HSA binding propensity of antibacterial drug chloroxine: multi-spectroscopic analysis, atomic force microscopy and docking simulation. Spectrochim. Acta A Mol. Biomol. Spectrosc..

[15] Martin SA (2009). Methotrexate induces oxidative DNA damage and is selectively lethal to tumour cells with defects in the DNA mismatch repair gene MSH2. EMBO Mol. Med..

[16] Najlah M (2016). Novel paclitaxel formulations solubilized by parenteral nutrition nanoemulsions for application against glioma cell lines. Int. J. Pharmaceutics.

[17] Chen D (2007). Clioquinol, a therapeutic agent for alzheimers disease, has proteasome-inhibitory, androgen receptor–suppressing, apoptosis-inducing, and antitumor activities in human prostate cancer cells and xenografts. Cancer Res..

[18] Daniel KG (2005). Clioquinol and pyrrolidine dithiocarbamate complex with copper to form proteasome inhibitors and apoptosis inducers in human breast cancer cells. Breast Cancer Res..

[19] Chou, D. & Martin, N. J.. CfDCPS, synergism UsGACPfQo, antagonism in drug combinations, IC50 tDo, ED50. ComboSyn Inc Paramus NJ. (2005).Accessed Dec 2020. https://www.combosyn.com/.

[20] Ding WQ, Liu B, Vaught JL, Yamauchi H, Lind SE (2005). Anticancer activity of the antibiotic clioquinol. Cancer Res..

[21] Tardito S (2012). Copper-dependent cytotoxicity of 8-hydroxyquinoline derivatives correlates with their hydrophobicity and does not require caspase activation. J. Med Chem..

[22] Zhai S (2010). Tumor cellular proteasome inhibition and growth suppression by 8-hydroxyquinoline and clioquinol requires their capabilities to bind copper and transport copper into cells. JBIC J. Biol. Inorg. Chem..

[23] Yu H, Lou JR, Ding W-Q (2010). Clioquinol independently targets NF-κB and lysosome pathways in human cancer cells. Anticancer Res..

[24] Xiao, M. et al. Comparison of different sample preparation methods for platinum determination in cultured cells by graphite furnace atomic absorption spectrometry. *PeerJ* **5**, e2873 (2017). 10.7717/peerj.2873 PMC5248575 28123908

[25] PubChem [Internet]. Bethesda (MD): National Library of Medicine (US), National Center for Biotechnology Information; 2004-. PubChem Compound Summary for CID 2722, Chloroxine. Available from: https://pubchem.ncbi.nlm.nih.gov/compound/Chloroxine.

[26] Corsello SM (2020). Discovering the anticancer potential of non-oncology drugs by systematic viability profiling. Nat. Cancer.

[27] Munro TA (2013). Selective kappa opioid antagonists nor-BNI, GNTI and JDTic have low affinities for non-opioid receptors and transporters. PLoS ONE.

[28] Redon CE (2010). Histone γH2AX and poly (ADP-ribose) as clinical pharmacodynamic biomarkers. Clin. Cancer Res..

[29] Tuller ER (2009). PPARα signaling mediates the synergistic cytotoxicity of clioquinol and docosahexaenoic acid in human cancer cells. Biochem. Pharmacol..

[30] Yang-Hartwich Y (2015). p53 protein aggregation promotes platinum resistance in ovarian cancer. Oncogene.

[31] Cooke SL, Brenton JD (2011). Evolution of platinum resistance in high-grade serous ovarian cancer. Lancet Oncol..

[32] Network CGAR. (2011). Integrated genomic analyses of ovarian carcinoma. Nature.

[33] Damia, G. & Broggini, M. Platinum resistance in ovarian cancer: role of DNA repair. *Cancers* **11**, 119 (2019). 10.3390/cancers11010119 PMC6357127 30669514

[34] Leroy B (2014). Analysis of TP53 mutation status in human cancer cell lines: a reassessment. Hum. Mutat..

[35] Domcke S, Sinha R, Levine DA, Sander C, Schultz N (2013). Evaluating cell lines as tumour models by comparison of genomic profiles. Nat. Commun..

[36] Bell D (2011). Integrated genomic analyses of ovarian carcinoma. Nature.

[37] Ghannam-Shahbari D (2018). PAX8 activates a p53-p21-dependent pro-proliferative effect in high grade serous ovarian carcinoma. Oncogene.

[38] Tym JE (2015). canSAR: an updated cancer research and drug discovery knowledgebase. Nucleic Acids Res..

[39] Basu A, Krishnamurthy S (2010). Cellular responses to cisplatin-induced DNA damage. J. Nucleic Acids.

[40] Gonzalez-Rajal, A., Hastings, J. F., Watkins, D. N., Croucher, D. R. & Burgess, A. Breathing New life into the mechanisms of platinum resistance in lung adenocarcinoma. *Front. Cell Dev. Biol.* **8**, 305 (2020). 10.3389/fcell.2020.00305 PMC7225257 32457904

[41] Bruno PM (2017). A subset of platinum-containing chemotherapeutic agents kills cells by inducing ribosome biogenesis stress. Nat. Med..

[42] Xie X, He G, Siddik ZH (2020). Cisplatin in combination with MDM2 inhibition downregulates Rad51 recombinase in a bimodal manner to inhibit homologous recombination and augment tumor cell kill. Mol. Pharmacol..

[43] Hine CM, Seluanov A, Gorbunova V (2008). Use of the Rad51 promoter for targeted anti-cancer therapy. Proc. Natl Acad. Sci. USA.

[44] Fransson Å (2016). Strong synergy with APR-246 and DNA-damaging drugs in primary cancer cells from patients with TP53 mutant high-grade serous ovarian cancer. J. Ovarian Res..

[45] Katsuyama M (2012). Clioquinol induces DNA double-strand breaks, activation of ATM, and subsequent activation of p53 signaling. Toxicology.

