# Supplementary material for: Chloroxine overrides DNA damage tolerance to restore platinum sensitivity in high-grade serous ovarian cancer
Source: Cell Death Dis. 2021 Apr 14;12(4):395. doi: 10.1038/s41419-021-03665-0 (PMC8047034; doi:10.1038/s41419-021-03665-0)
Supplement: Supplementary file 1 — Supplementary Figures and Legends [file 41419_2021_3665_MOESM1_ESM.docx]

**Supplementary Figures**

Supplementary Figure S1 – Effect of chloroxine, carboplatin and cisplatin on the viability of OVCAR4 (sensitive) and Ov4Carbo (resistant) ovarian cancer cells after 72h treatment

Supplementary Figure S2 – Synergy between chloroxine and cisplatin in paired OVCAR4 (sensitive)/OV4Cis (resistant) and COV318 (sensitive)/COV318Cis (resistant) ovarian cancer cells

Supplementary Figure S3 – Synergy between chloroxine (Chl. 10 µM) and carboplatin in Ov4Carbo (resistant) single cell clones 4 (Ov4Carbo4) and 7 (Ov4Carbo7)

Supplementary Figure S4 – Analysis of CuCl2 mediated synergy in OVCAR4 and Ov4Carbo cells

Supplementary Figure S5 – Analysis of OPRK1 mediated synergy in OVCAR4 and Ov4Carbo cells

Supplementary Figure S6 – Subcellular analysis of carboplatin and chloroxine induced DNA damage overtime in OVCAR4 (sensitive) ovarian cancer cells

## Figures and legends


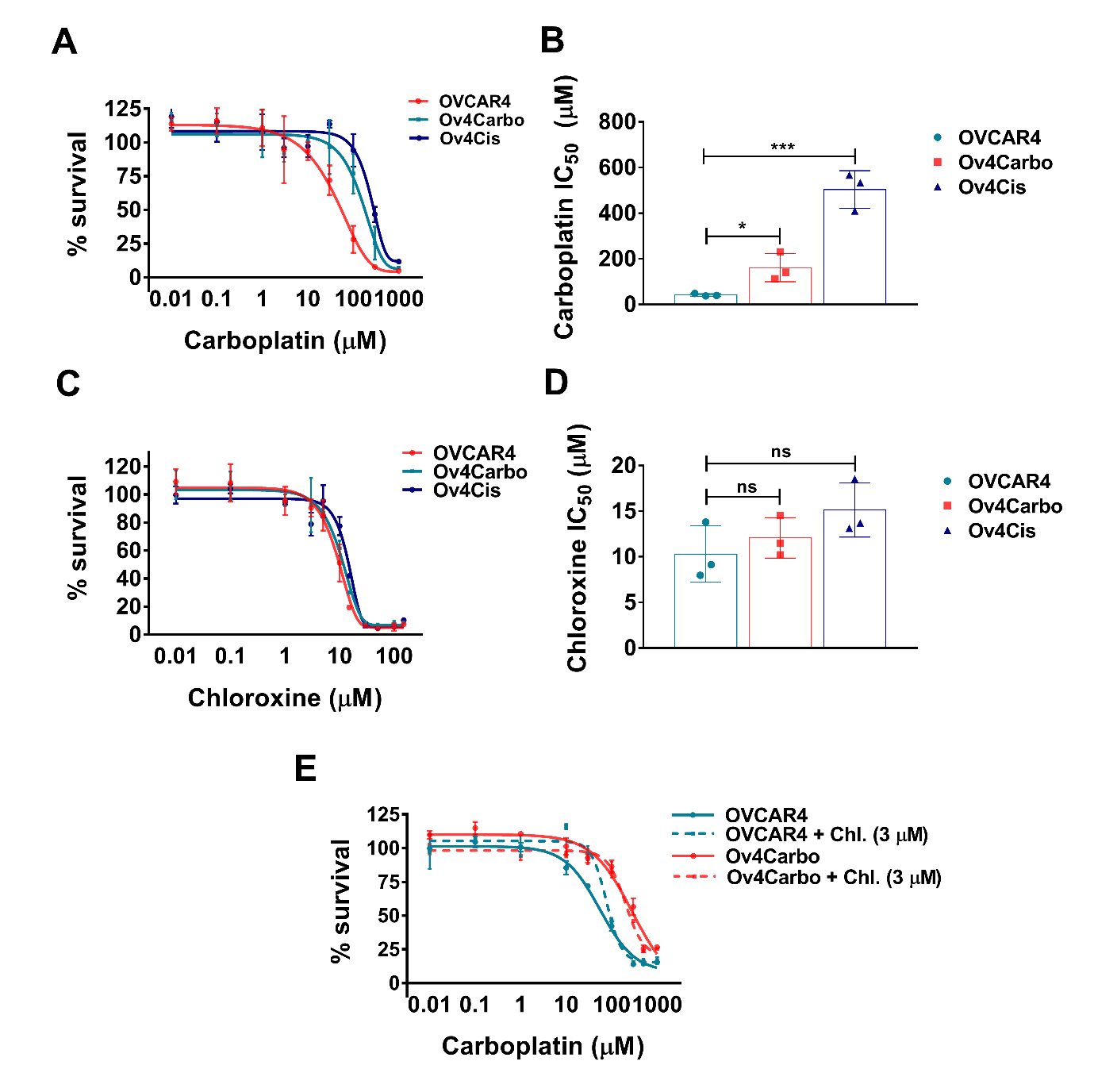


**Figure S1. Effect of carboplatin and chloroxine on the viability of OVCAR4 and Ov4Carbo HGSC cells after 72h treatment. A-B)** Dose-response and IC_50_ plot for carboplatin; **C-D)** Dose-response and IC_50_ plot for chloroxine; **E)** Dose-response to carboplatin + chloroxine (3 μM). Cell viability was determined using CellTiter-Glo®. IC_50_ was calculated using GraphPad Prism v.8.3.0. n=3 biological repeats, mean ± s.d., unpaired t-test, **P*<0.05 ****P*<0.001, ns: not significant.


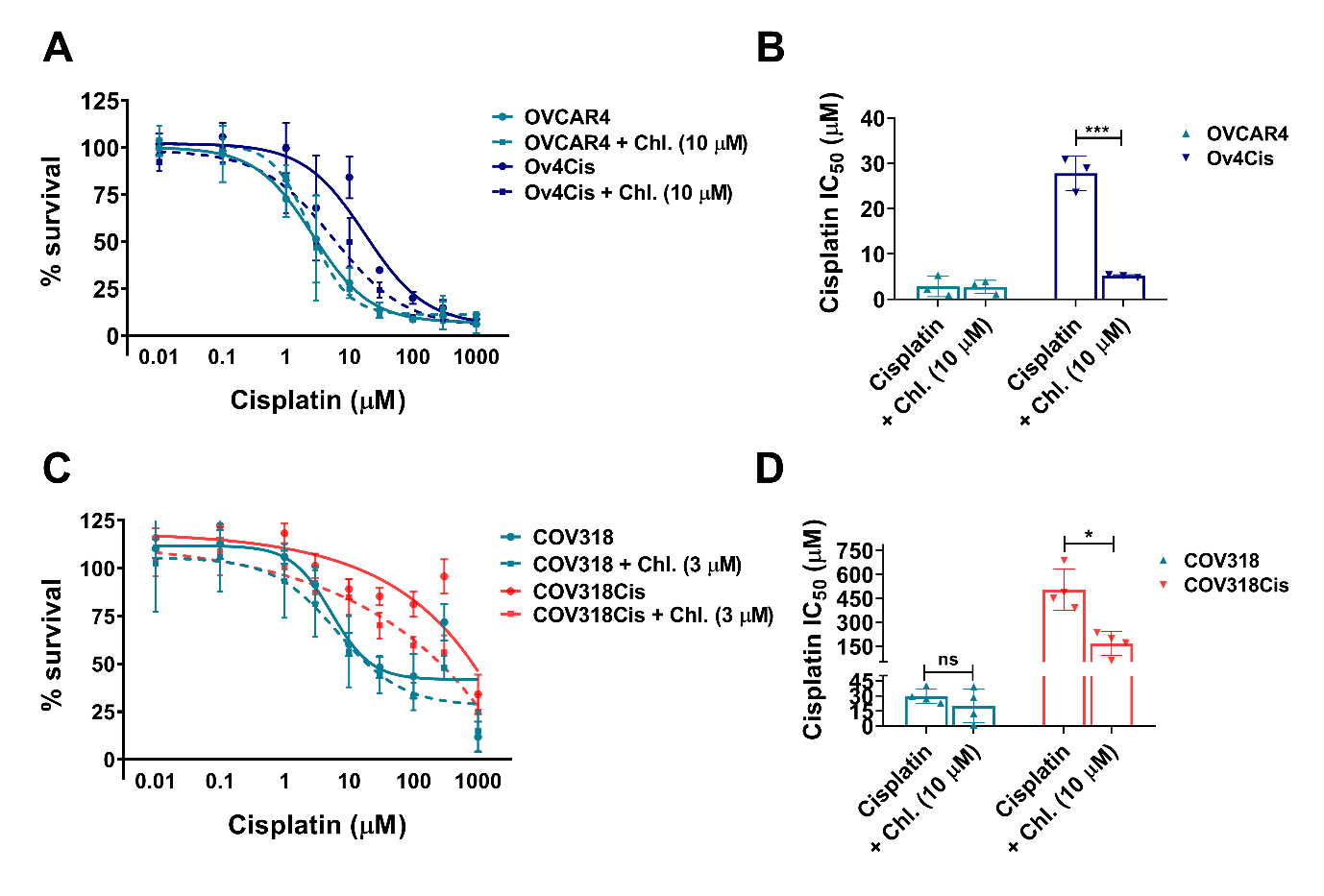


**Figure S2. Synergy between chloroxine and cisplatin in paired OVCAR4 (sensitive)/OV4Cis (resistant) and COV318 (sensitive)/COV318Cis (resistant) ovarian cancer cells. A-B)** Dose-response and IC_50_ plot for cisplatin + chloroxine (10μM) in OVCAR4 cells; **C-D)** Dose-response and IC_50_ plot for cisplatin + chloroxine (3μM) in COV318 cells; Cell viability was determined using CellTiter-Glo®. IC_50_ was calculated using GraphPad Prism v.8.3.0. n=3 biological repeats, mean ± s.d, unpaired t-test, **P*<0.05, ****P*<0.001


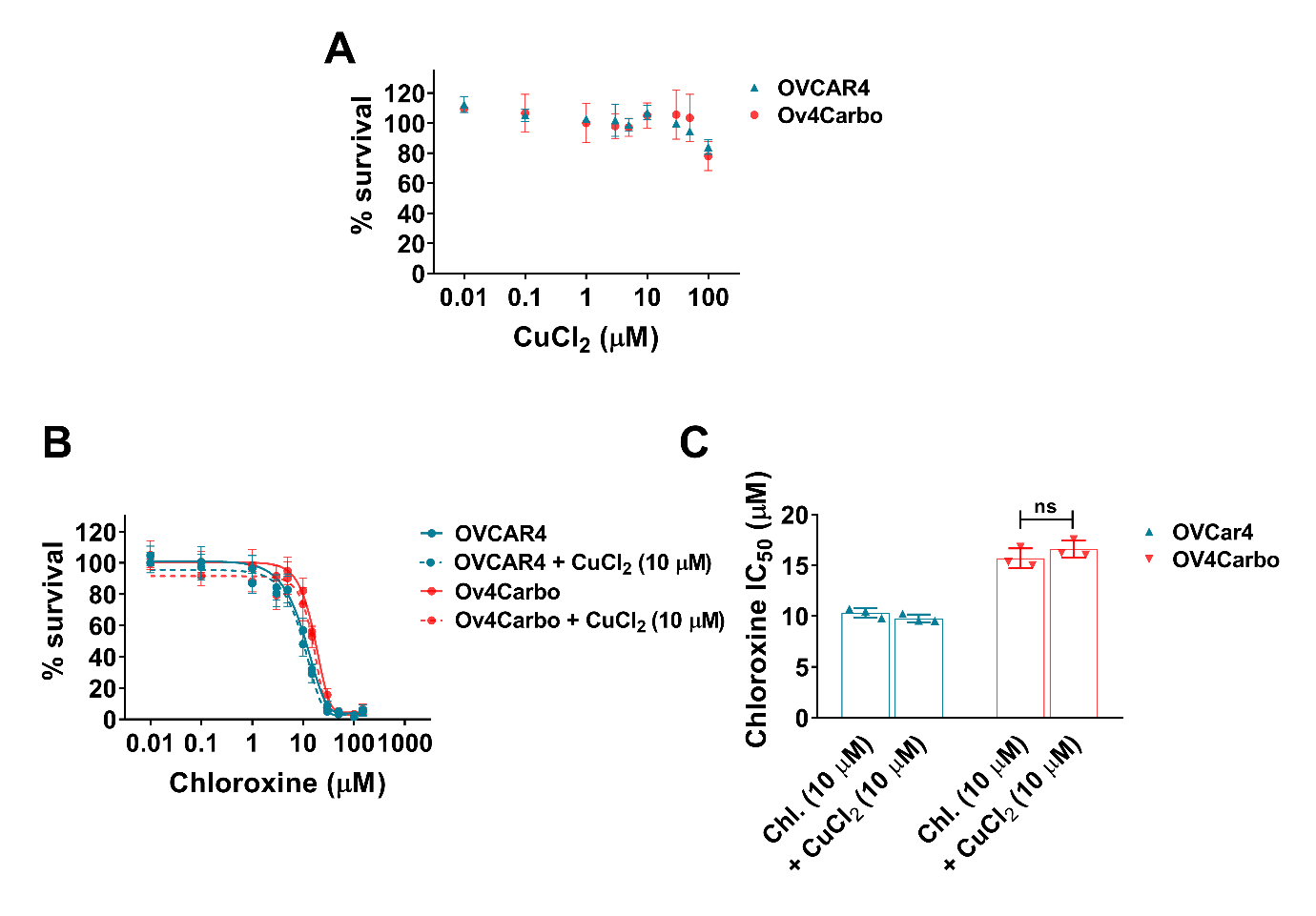


**Figure S3. Analysis of CuCl_2_ mediated synergy in OVCAR4 and Ov4Carbo cells. A)** Dose-response to increasing concentrations of CuCl_2_, indicating no cell death; **B)** Dose-response and IC_50_ plot for chloroxine in media enriched with CuCl_2_ (10μM) for 48h**.** Cell viability was determined using CellTiter-Glo®. IC_50_ was calculated using GraphPad Prism v.8.3.0. Mean ± s.d, n=3 biological repeats, unpaired t-test, ns: not significant.


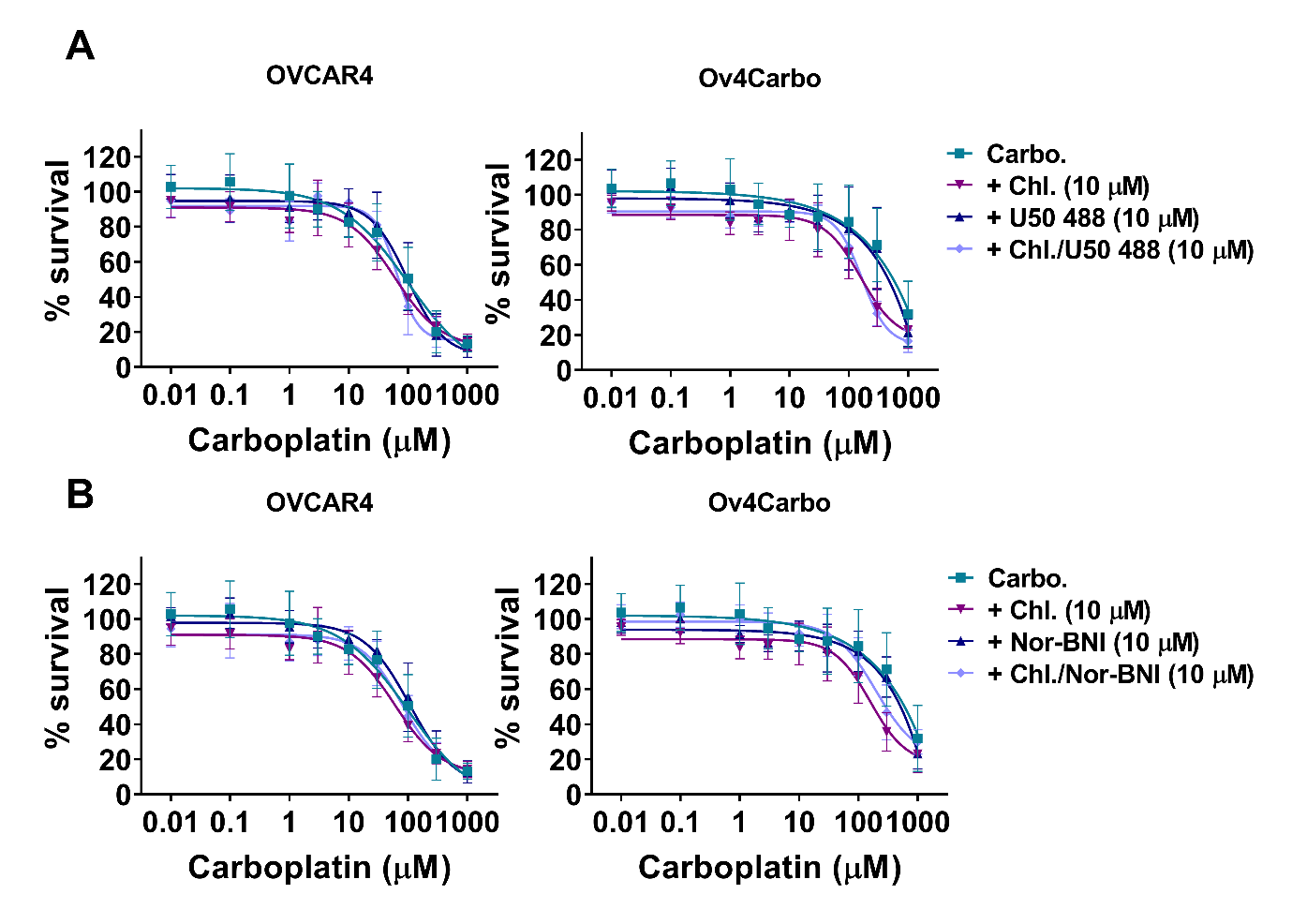


**Figure S4. Analysis of OPRK1 mediated synergy in OVCAR4 and Ov4Carbo cells.** Dose-response curves for carboplatin + chloroxine (10μM) containing **A)** OPRK1 agonist (U50-488, 10μM) and B**)** OPRK1 antagonist (nor-BNI, 10μM. Cell viability was determined using CellTiter-Glo®. IC_50_ was calculated using GraphPad Prism v.8.3.0. n=8 biological repeats, mean ± s.d.


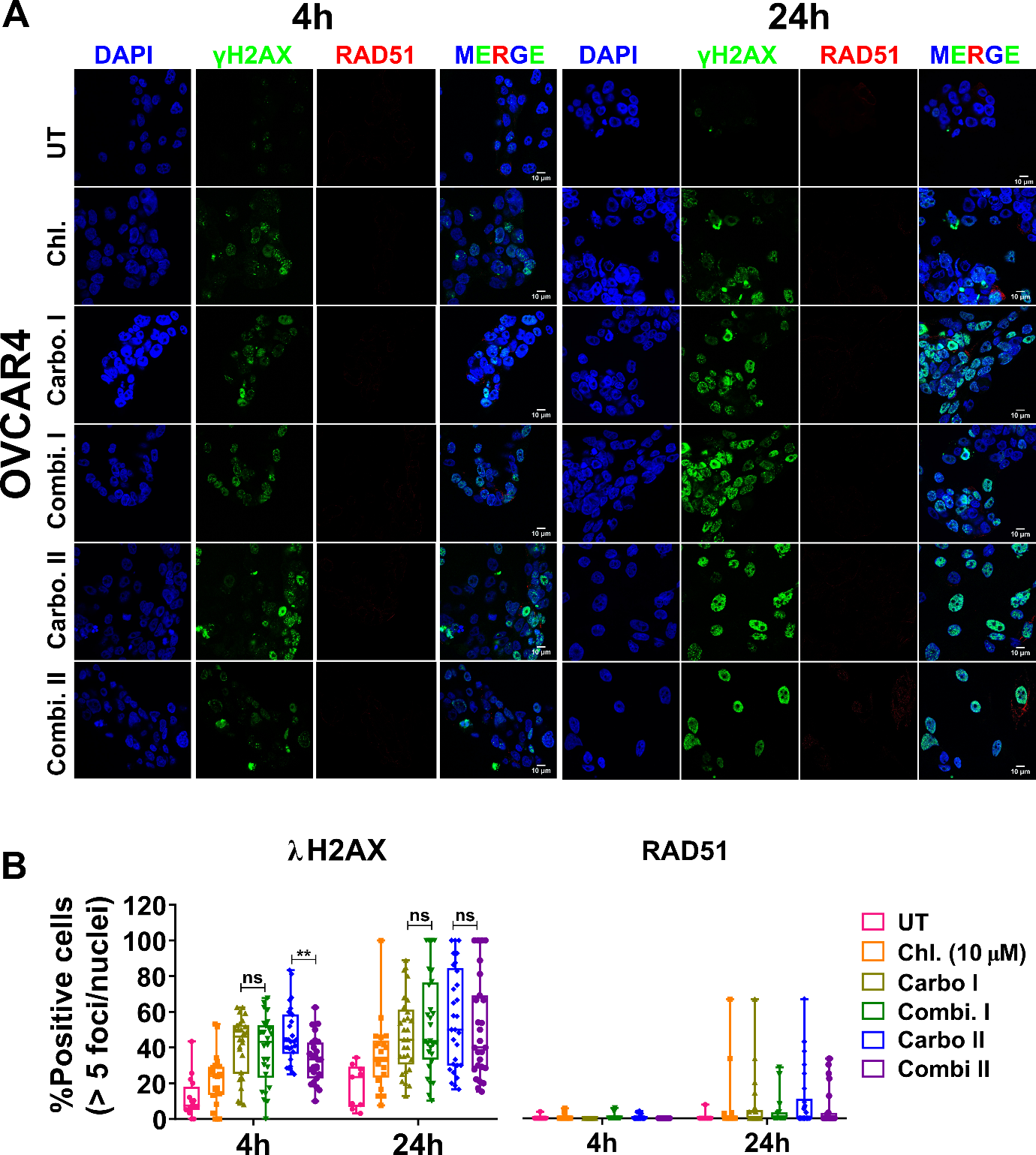


**Figure S5. Subcellular analysis of carboplatin and chloroxine induced DNA damage overtime in OVCAR4 (sensitive) ovarian cancer cells.** A) Representative immunofluorescent staining of γ-H2AX and RAD51 and B) foci quantification after 4 and 24h treatment. Cells were treated with chloroxine (10 μM), carboplatin I (50 μM), combi. I (chloroxine 10 μM + carboplatin 50 μM), carbo II (100 μM) or combi. II (chloroxine 10 μM + carboplatin 100 μM). Untreated cells were used as control. Quantification was expressed as percentage of cells that showed more than 5 foci/nuclei at each time-point. Data shows mean ± s.d, of individual ROI of duplicate coverslips. At least 50 cells were quantified for each independent experiment (4h, n=3 and 24h, n=4 biological repeats). Two-way ANOVA with Tukey post-*hoc* test: ***P*<0.01 and *P*>0.05 (ns).


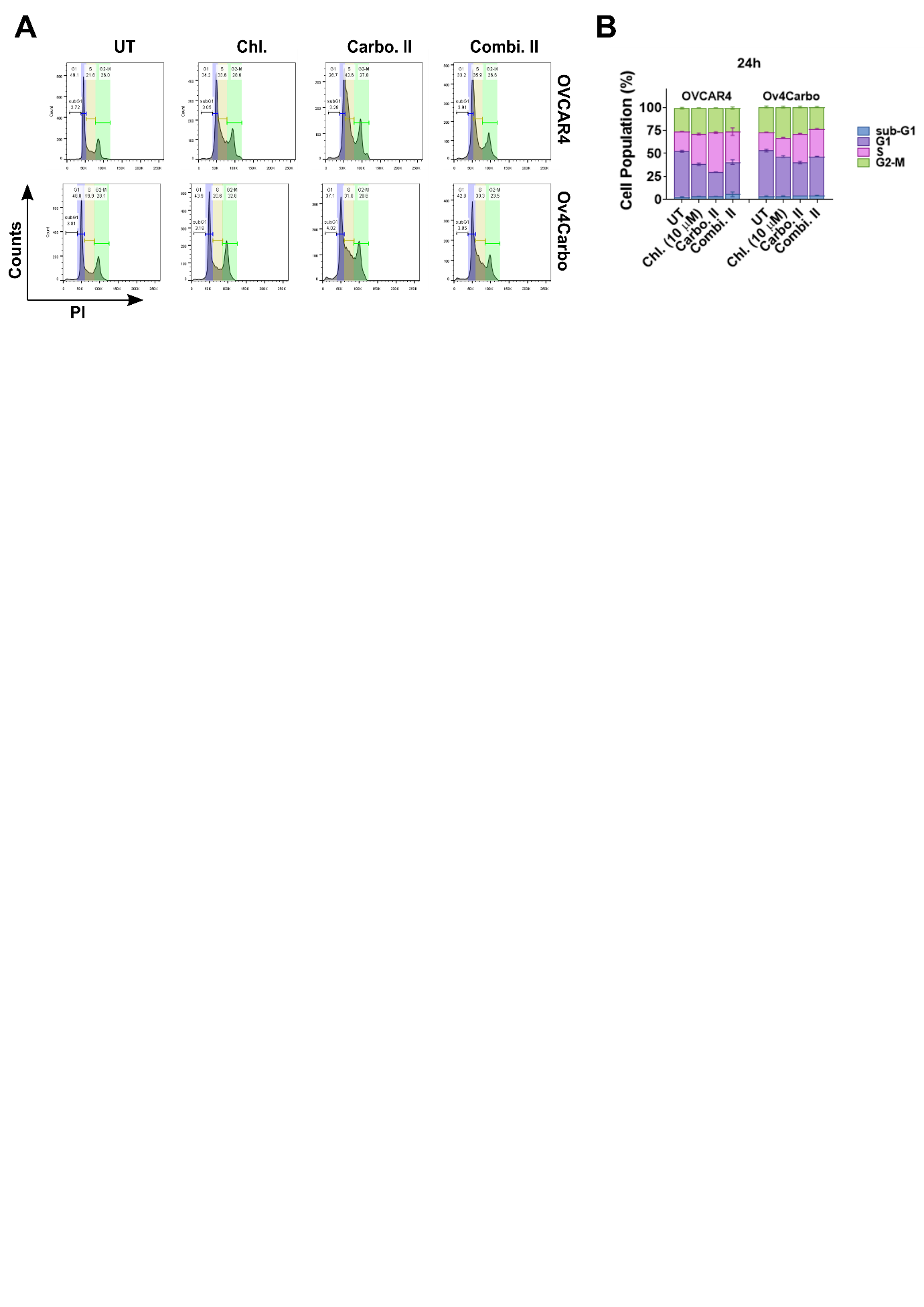


**Figure S6**. **Analysis of cell cycle and cell death in OVCAR4 (sensitive) and OV4Carbo (resistant) ovarian cancer cells. A)** Representative histograms 24h post-treatment; **B)** cell cycle distribution and (top) and percentage of sub/G1 apoptotic cells (bottom). Cells were treated with either chloroxine (10μM), carbo II (100μM) or combi. II (chloroxine 10μM + carboplatin 100μM). Untreated cells were used as control. Data shows mean ± s.d, and percentage of G1 (2n), S and G2/M (4n) fraction population (n=2 biological repeats).
