# Supplementary material for: Chloroxine overrides DNA damage tolerance to restore platinum sensitivity in high-grade serous ovarian cancer
Source: Cell Death Dis. 2021 Apr 14;12(4):395. doi: 10.1038/s41419-021-03665-0 (PMC8047034; doi:10.1038/s41419-021-03665-0)
Supplement: Supplementary file 3 — Supplementary Tables and Legends [file 41419_2021_3665_MOESM3_ESM.docx]

**Supplementary Tables**

**Suppl Table 1**

See attached excel file entitled Suppl Table 1

**Supplementary Table 1: Log2-surviving fraction of initial drug screen**

Complete list of log2-surviving fraction for each well of the initial compound library screen in OVCAR4 and OvCarbo cells. Results for all experimental conditions are shown including control-treated wells.

| Drug Name | OVCAR4 | | Ov4Carbo | |
| --- | --- | --- | --- | --- |
|  | Drug alone | Drug + carboplatin | Drug alone | Drug + carboplatin |
| Triamcinolone | -0.08716 | -0.87706 | 0.188061 | -10.5611 |
| Sulbactam | -0.9644 | -1.01162 | 0.278896 | -9.85507 |
| Sarafloxacin HCl | -0.17874 | -0.77007 | 1.291311 | -9.66169 |
| Fluticasone propionate | 0.046324 | 0.663494 | 0.603676 | -9.51073 |
| Pyrimethamine | -1.11673 | -1.21671 | -0.59847 | -9.14999 |
| Ginkgolide A | -0.95463 | 0.721772 | 0.24433 | -8.20249 |
| Lapatinib | -0.86862 | 1.347388 | -1.05534 | -6.03331 |
| Flubendazole | -0.3866 | 0.695168 | -0.85866 | -5.94302 |
| Diethylstilbestrol | -0.05333 | -0.00788 | 0.094976 | -4.68921 |
| **Chloroxine** | **-0.62565** | **-3.6396** | **0.120017** | **-4.17503** |
| Amlodipine | -0.87412 | -1.69659 | -0.99266 | -2.99842 |

**Suppl Table 2:**

**Supplementary Table 2**: **Hit drugs identified in compound library screen.**

log2-surviving fraction (s.f.) is shown following treatment of OVCAR4 and Ov4Carbo with the indicated drugs. Drug alone = s.f. in wells treated with compound library drug compared to vehicle treatment. Drug + carboplatin = s.f. in wells treated with compound library drug + carboplatin compared to carboplatin alone.
